# Bromodeoxyuridine Labelling to Determine Viral DNA Localization in Fluorescence and Electron Microscopy: The Case of Adenovirus

**DOI:** 10.3390/v13091863

**Published:** 2021-09-18

**Authors:** Gabriela N. Condezo, Carmen San Martín

**Affiliations:** Department of Macromolecular Structures, Centro Nacional de Biotecnología (CNB-CSIC), 28049 Madrid, Spain

**Keywords:** bromodeoxyuridine, DNA labeling, adenovirus, fluorescence microscopy, electron microscopy

## Abstract

The localization of viral nucleic acids in the cell is essential for understanding the infectious cycle. One of the strategies developed for this purpose is the use of nucleotide analogs such as bromodeoxyuridine (BrdU, analog to thymine) or bromouridine (BrU, analog of uridine), which are incorporated into the nucleic acids during replication or transcription. In adenovirus infections, BrdU has been used to localize newly synthesized viral genomes in the nucleus, where it is key to distinguish between host and viral DNA. Here, we describe our experience with methodological variations of BrdU labeling to localize adenovirus genomes in fluorescence and electron microscopy. We illustrate the need to define conditions in which most of the newly synthesized DNA corresponds to the virus and not the host, and the amount of BrdU provided is enough to incorporate to the new DNA molecules without hampering the cell metabolism. We hope that our discussion of problems encountered and solutions implemented will help other researches interested in viral genome localization in infected cells.

## 1. Introduction

The need to localize viral nucleic acids in infected cells has driven the development of different strategies: stains, antibodies against nucleic acids, radioactive labeling of nucleotides, in situ hybridization, or labeling of nucleotide analogs [1]. Each one of these methods has its pros and cons. Stains or antibodies against nucleic acids are easy to use; however, with these methods, it is not possible to distinguish between host and viral nucleic acids. Radioactive labeling is highly sensitive and accurate, but it has been substituted by other earth-friendly strategies. In situ hybridization uses DNA or RNA probes labeled with haptens such as biotin, digoxigenin or acetoxyacetylaminofluorene, or enzymatic labels such as biotin-streptavidin. This methodology allows accurate localization of specific segments of viral genomes, although it takes time to design probes and standardize hybridization conditions [2]. Nucleotide analogs with modifications to facilitate their detection can be used to label newly synthesized nucleic acids. Some examples are dUTP labeled with fluorescent labels, or fluorescent pteridine nucleoside analogues [1]. EdC (5–ethynyl 2-deoxy cytidine) or EdU (5-ethynyl-2′–deoxyuridine) [3,4] are nucleoside analogs used in click chemistry reactions. Their reaction with copper(I)-catalyzed azide-alkyne allows DNA visualization without sample denaturation [4]. A recent development is the ANCHOR3/ParB technology, derived from the bacterial partitioning system ParB-*parS*. ANCHOR3/ParB allows *in vivo* labeling of DNA molecules carrying several *parS* sites by binding and oligomerization of protein OR3/ParB fused to fluorescent molecules. This system requires genetic modification of the viral genome to include the *parS* sites. Expression of the fluorescently tagged OR3/ParB can also be obtained by genetic modification of the viral genome, or by transfection of the host cell [5].

Bromodeoxyuridine (BrdU) and bromouridine (BrU) can be included within the group of techniques using incorporation of nucleotide analogs, although they do not have modifications that help their detection; in this case, specific antibodies against the analogs are used. BrdU is an analog of thymine, and BrU is an analog of uridine; they are used for labeling DNA and RNA, respectively. Radioimmunoassays show that the substituent (bromine) on uridine C5 is essential for the antibody reactivity; the region around pyrimidine C4 is also important for antibody recognition [6]. Taking this into account, it seems logical that several commercial companies offer BrdU antibodies that also recognize BrU [7]. To label viral nucleic acids, these compounds are added to the medium during the infection, so that they are incorporated into viral genomes during the replication. A denaturing treatment is required for the localization of dsDNA and dsRNA, to expose the incorporated analogs to the antibodies. As early as 1990, when low temperature dehydration and embedding protocols facilitated the development of immunoelectron microscopy, BrdU was used to follow different DNA species in adenovirus (AdV)-infected cells [8,9]. BrdU has also been used to localize AdV DNA in fluorescence microscopy [10,11,12,13]. Short pulses with BrdU added shortly before the end of the required post-infection time allow visualization of active replication sites [8,9,10,11,12,13]. Longer pulses, after which BrdU-containing medium is removed and the infection allowed to proceed for a longer time, have been used to analyze genome migration [8,9]. Virions with BrdU labeled genomes are generated by maintaining BrdU in the medium throughout the infection [14]. These can be used for entry studies.

Adenoviruses are complex, icosahedral non-enveloped viruses with virions composed by ~15 different proteins and the linear dsDNA genome (~35 kbp in the human AdV). AdV replication and assembly occur in the nucleus, presenting a challenge to distinguish between viral and host DNA. During the AdV infection, the nucleus undergoes drastic changes to harbor AdV replication compartments (or replication centers, RC), which function as platforms for viral DNA replication and gene expression. These compartments consist of cellular and viral macromolecules [15]. The AdV replication cycle is divided in two phases, early and late, separated by the onset of viral DNA replication [16,17]. For human adenovirus type 5 (Ad5), the infectious cycle is completed after 24 to 36 h. During the early phase, the viral genome (bound to core proteins) just arrived at the nucleus interacts with the host proteins. Among these interactions stand out: protection of the viral genome from cellular DNA damage response; inhibition of the cellular antiviral response; start and regulation of the viral transcription and replication. All this complex system of interactions leads to the formation of early replicative sites (ERS), where early viral gene expression occurs (starting from ~1–2 h post-infection (hpi)) (reviewed in [18]). ERS contain viral ssDNA, dsDNA, as well as viral spliced and unspliced mRNAs (reviewed in [15]). Newly synthesized AdV DNA is first detected at ~6–8 hpi [18]. The viral replication rate increases between 8 and 16 hpi, after which it decreases [19].

**Figure 1:**
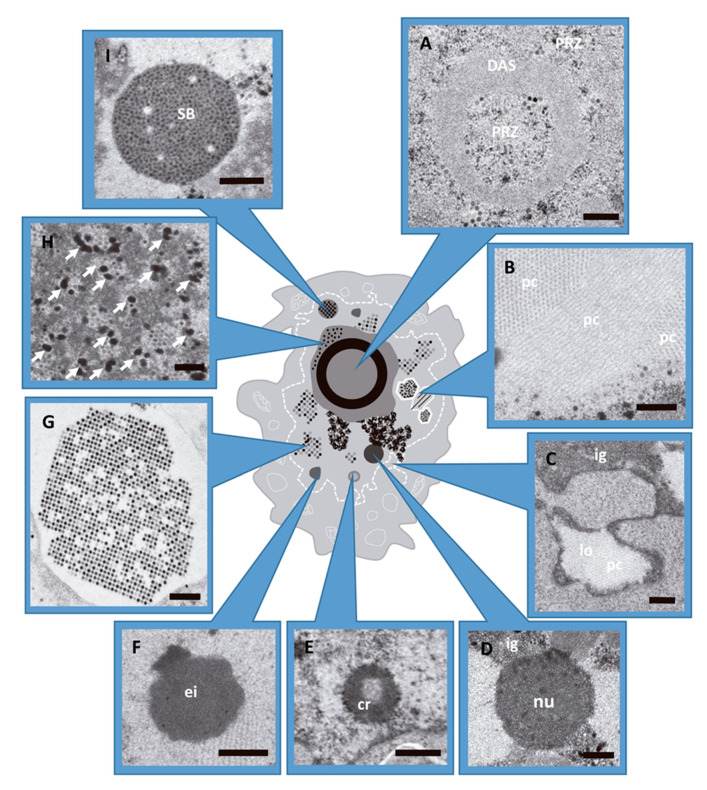
AdV induced alterations in the host cell nucleus during infection. HEK293 cells infected with AdV and embedded in Epon (see Methods). (**A**) Peripheral replicative zone (PRZ) and ssDNA accumulation site (DAS). Cell infected with Ad5GL at 48 hpi. (**B**) Viral protein crystal (pc), cross and longitudinal section. Ad5GL infection at 48 hpi. (**C**) Lobule (lo) and cluster of interchromatin granule (ig). Ad5/FC31 infection at 24 hpi. (**D**) Nucleolus (nu) from cell infected with Ad5GL at 48 hpi. (**E**) Compact ring (cr). Ad5/FC31 infected cell at 24 hpi (**F**) Electron-dense inclusion. Ad5/FC31 infection at 48hpi (**G**) Section of a paracrystalline array of virus particles (cell infected with Ad2 *ts1* at 48 hpi). (**H**) Electron-dense granules (arrows) located within the PRZ. Ad5/FC31 infection at 48 hpi. (**I**) Speckled body found in Ad5/FC31 infection at 56 hpi. (**A**–**D**,**F**,**H**) Bar: 400 nm. (**E**,**G**,**I**) Bar: 500 nm. Images obtained by the authors during a survey of morphological changes in HEK293 cells infected with different Ad5/Ad2 variants. Panels D and I modified from [27].

As the infection progresses (from 17 hpi aproximately), ERS give way to the generation of new compartments: the ssDNA accumulation site (DAS) where DBP (AdV ssDNA binding protein) can be detected, together with a large number of single stranded replicative intermediates; and a peripheral replicative zone (PRZ) where viral dsDNA is accumulated and there is continuous replicative activity [20,21,22]. This zone is often found surrounding the DAS, which has intermittent replicative activity. Later in infection (close to 24 hpi), a single large viral genome storage site is developed. This is the main site of storage for non-replicating dsDNA viral genomes [8,20,21,22,23]. At intermediate times post-infection (17–20 hpi), the DAS forms a ring-like structure [24,25,26] with the PRZ located adjacent to both the inner and outer borders of this ring (Figure 1A) [11,21]. In HEK293 cells, the PRZ is the only nuclear region where most representative players of AdV morphogenesis have been localized, strongly suggesting that this is the AdV assembly factory [27].

At late times (~24 hpi), virus particles and protein crystals formed by penton base and fiber start to appear [28,29] (Figure 1B). At 36 hpi, particle production reaches its maximum [30] (Figure 1G) and the cell morphology is extensively altered. The nuclear membrane adopts an irregular outline or proliferates abnormally forming lobes (Figure 1C), and cell chromatin is pushed towards the nuclear border to be excluded from viral RCs [31,32,33]. Nucleoli are compacted (Figure 1D) and many structures containing viral components appear: compact rings (RNA and packaging protein IVa2) (Figure 1E), electro-dense (Figure 1F) and electron-lucent inclusions (hexon, penton, IVa2, L1 52/55 kDa, and IX) [27,34,35,36,37,38,39]. Electron-dense granules (Figure 1H) containing viral RNA, protein VII and viral genomes were found in the clusters of interchromatin granule and in the PRZ [9,27]. Finally, speckled bodies (SB) accumulate genomes and core proteins which have failed to be encapsidated (Figure 1I) [27].

Setting up BrdU localization assays requires consideration of the particular experimental goal (what do we want to localize), the previously known dynamics of the infectious cycle, and the visualization method to be employed (fluorescence or electron microscopy). Nucleotide analogs have to be added at the proper time, and in the proper conditions, to allow the infection to proceed in a state as close as possible to the natural one, while providing enough signal for localization of the desired target. We have previously used BrdU in combination with immunofluorescence and immunoelectron microscopies to localize AdV assembly factors (genome, packaging proteins, capsid and core proteins) in the nucleus of the infected cell. This work allowed us to propose that AdV assembly takes place at the PRZ, and also revealed the presence of assembly intermediates and failed products consistent with a concurrent assembly and packaging model [27]. Here, we present a detailed account of the process used to arrive at the final protocol published in [27], focusing on the methodological aspects of AdV genome labeling using BrdU, comparison with conditions used in previous works, problems found and solutions implemented.

## 2. Materials and Methods

### 2.1. Viruses and Cells

The viruses used in this study were: Ad5GL, a nonreplicative, E1 deleted, structurally wild type (wt) Ad5 variant carrying the GFP and firefly luciferase genes (designated by the suffix “GL”) [40]. Ad5/FC31 is a nonreplicative, E1 and E3 deleted Ad5 variant that contains an *attB/attP* insertion flanking the packaging sequence motif Ψ, and a GFP cassette following Ψ [30]. Ad5/FC31 is a delayed packaging mutant: there is a 20 h delay in its viral cycle, and large amounts of genome-less capsids are produced [30,41]. Adenovirus type 2 (Ad2) *ts1* is replicative at 32 °C and nonreplicative at 39 °C. This virus contains a thermosensitive mutation in the protease gene [42]. At 39 °C it produces only young virions. 

HEK 293 are human embrionic kidney cells transformed with sheared Ad5 DNA [43]. These cells are ideal for propagation of E1 deleted Ad5 variants. HEK293 cells were propagated in Dulbecco’s modified Eagle’s medium (DMEM, Sigma Cat# D6429) supplemented with 10% fetal bovine serum (FBS, Biological Industries Cat# 04-001-1A), 10 units-10 µg/mL penicillin-streptomycin (Sigma Cat# P4333), 0.05 mg/mL gentamicin (Sigma Cat# G1397), 4 mM L-Glutamine (MERCK Cat# 3520, Kenilworth, NJ, USA) and 1X non-essential amino acid solution (Sigma Cat# M7145), and maintained at 37 °C in a humidified incubator with 5% CO_2._ During the infection, FBS concentration was 2%.

### 2.2. Immunofluorescence Microscopy

Cover glasses (diameter: 12 mm) were incubated on poly-L-lysine (Sigma-Aldrich Cat # P4707, St. Louis, MO, USA) for 30 min at 37 °C, then placed in 24-well culture plates (Thermo Scientific Nunc^TM^ Cat# 142475, Waltham, MA, USA) and washed with PBS (137 mM NaCl, 2.7 mM KCl, 10 mM Na_2_HPO_4_, 1.8 mM KH_2_PO_4_ pH 7.4). HEK293 cells at 70–80% confluence were diluted 1:5 in DMEM and seeded on the cover glasses. After 24 h, cells were infected with AdV (virus variant and multiplicity of infection (MOI) according to the experiment) in 200 µL inocula. Infections were synchronized by incubating the cells for 30 min at 4 °C and then 30 min at 37 °C. Then, the inocula were removed and DMEM was added. For BrdU labeling, the compound (25 µg/mL BrdU, Sigma Cat#B5002-1G) was added to the medium of uninfected or infected cells and incubated at 37 °C during the desired time in accordance with the experiment goal.

At 36 hpi, the medium was removed and the cells were washed with PBS. A solution of 4% paraformaldehyde in PBS was added to the cells for 10 min. The fixed samples were washed three times with saponin 1% in PBS (3 × 5 min), then subjected to DNA denaturing treatment: 1N HCl during 10 min at 4 °C, followed by 2N HCl during 10 min at room temperature, and finally 20 min at 37 °C in the same solution. Borate buffer (4 g NaOH; 23.5 g borate acid to 500 mL pH 8.2) was added to neutralize during 12 min at room temperature (Abcam protocol). Samples were rinsed with saponin 1% in PBS (3 × 5 min), and incubated with 1% saponin and10% FBS during 10 min. Then, the cells were rinsed with saponin 1% in PBS (2 × 5 min), and incubated with primary antibodies: rat anti-BrdU (abcam Cat # ab6326, dilution 1:250), mouse anti-GFP (Sigma-Merck # 11 814 460 001, dilution 1:200) or mouse anti Ad5 DBP monoclonal (dilution 1:20) [44] in 1% saponin and 2% FBS in PBS for 45 min. Controls included: mock infected cells incubated with primary antibody in the same conditions as infected cells, and incubations without primary antibody for both mock and infected cells. After three more rinses, incubation with secondary antibodies was carried out in darkness. The secondary antibodies: Alexa Fluor^®^594 Goat Anti-Rat (Invitrogen # A-11007) or Alexa Fluor^®^555 Goat Anti-Rat (Invitrogen # A-21434), Alexa Fluor^®^488 Goat Anti-Mouse (Invitrogen # A-11029) and Pacific Blue^TM^ Goat Anti-Mouse (Invitrogen # P-31582) were diluted 1:500 in 1% saponin and 2% FBS in PBS. Samples were rinsed 3 times with PBS before adding DAPI (Sigma Cat#32670) for DNA staining (15 min, dilution 1:200 in PBS). After a final rinse with PBS, cover glasses were mounted on glass slides using ProLong (Invitrogen Cat# P36930) drops (4 µL). The antifade reagent was allowed to dry overnight before sample observation. All incubations were carried out at room temperature. Images were taken using a confocal multispectral Leica TCS SP5 system.

For double labeling assays, the anti-GFP and anti-BrdU antibodies, or the anti-BrdU and anti-DBP antibodies were used together in the same incubation. The secondary antibodies were incubated together. The controls for double BrdU/DBP labelling were: mock and infected cells without primary antibodies, mock cells with the two primary antibodies, and infected cells with only anti-DBP without HCl treatment. Immunofluorescence image analyses were carried out with Image J [45].

### 2.3. Conventional Electron Microscopy of Infected Cells

HEK293 cells were grown in a p100 culture plate to 70% confluence, then infected with Ad5/FC31 with MOI = 5. At the desired time post infection, the medium was removed and the cells were fixed with 2% glutaraldehyde and 1% tannic acid in 0.4 M HEPES, pH 7.2 during 1.5 h at room temperature. Dehydration and embedding in Epon resin (812 Epon Embedding Kit, Electron Microscopy Sciences) was performed as described [46]. Ultrathin sections (~70 nm) were obtained using a Leica EM UC6 Ultramicrotome and collected on Formvar-coated nickel grids (200 mesh, 0.25% Formvar). Sections were stained with saturated uranyl acetate for 25 min, floated on 4 drops of milli-Q water, stained on 0.2% lead citrate drops (in 0.4% NaOH) for 1 min, and washed in 4 drops of milli-Q water. The grids were examined in a JEOL JEM 1230 transmission electron microscope at 100 kV.

### 2.4. Immunoelectron Microscopy

BrdU labeling of newly synthesized DNA was carried out as described for immunofluorescence, except for HEK293 cells grown in p100 culture plates without cover glasses, and using different compound concentrations as described in Results. Infected and control cells were fixed with 4% paraformaldehyde in PBS for 10 min at room temperature, after medium removal. After rinsing three times with PBS, glycerol was added drop by drop up to 15% concentration. After 15 min at 4 °C, glycerol was increased to 30%. After 15 more minutes at 4 °C, the cells were harvested and centrifuged for 10 min at 5000 rpm. The pellets were placed on small squares (0.2 × 0.2 cm) of Whatman paper and frozen by plunge freezing in liquid ethane using a Leica CPC plunger. Freeze substitution was carried out in a Leica EM automatic freeze substitution system (AFS) as described [27]. Ultrathin sections were collected as indicated in the previous section. 

For BrdU immunolabeling, sections were treated with 0.2 mg/mL proteinase K (Roche Cat# 3115879) for 15 min at 37 °C, then washed with milli-Q water and denatured with 2N HCl for 25 min. After several (~4) rinses in milli-Q water, the grids were placed on TBG (30 mM Tris-HCl pH 8, 150 mM NaCl, 0.1% BSA and 1% gelatin) drops with the sections in contact with the solution for 10 min, and then incubated with the same Rat anti-BrdU antibody used for immunofluorescence (dilution 1:25) in TBG for 30 min. Controls included: mock infected cells incubated with primary antibody in the same conditions as infected cells; incubations without primary antibody for both mock and infected cells; as well as mock and infected cells without BrdU incorporation labeled against BrdU. The grids were washed 3 times with PBS, and then floated on 4 drops of TBG (5 min per drop). Incubation with 15 nm Gold-conjugated Goat Anti-Rat (BB international #EM-GAT15) diluted 1:40 in TBG was carried out for 30 min. Then, grids were washed 3 times with PBS and milli-Q water, and stained with saturated uranyl acetate as described in the previous section. Lead citrate was not used in this case. All incubations were carried out at room temperature. The grids were examined in a JEOL JEM 1230 transmission electron microscope at 100 kV.

## 3. Results

For our AdV genome localization assays, we used infected cells at late times post-infection (36 or 48 hpi), because at this point the AdV factory is established, cell modifications induced by the virus are clearly visible, and differences between Ad5GL and Ad5/FC31 related to the packaging delay were evident [27]. The use of Ad5 variants with a GFP cassette facilitated identification of infected cells. Confocal fluorescence microscopy (FM) was used first, and the best BrdU incorporation and labeling conditions found were later evaluated and adapted for immunoelectron microscopy. Electron microscopy (EM) provides increased detail on the ultrastructure of the infected cells, while FM provides a more efficient way to test many different experimental conditions. Infection, labeling and observation of up to 16 FM samples can be completed in one week, while EM sample preparation, freeze-substitution, sectioning, labelling and finally imaging takes at least four weeks, or more if the results require to prepare and section more blocks. In freeze substitution the space for sample processing is limited by the AFS design, which allows only 16 blocks to be processed at the same time (i.e., eight different samples in duplicate). Additionally, FM allows imaging of many complete cells in a brief period of time, while EM observation of many cells at high magnification is a more time consuming process. A summary of the conditions described in the following sections is presented in Table 1. Table 2 lists the problems we observed, their possible reasons, and the solutions found.

### 3.1. Optimizing the Pulse Time: Labeling with Short BrdU Pulses

**Figure 2:**
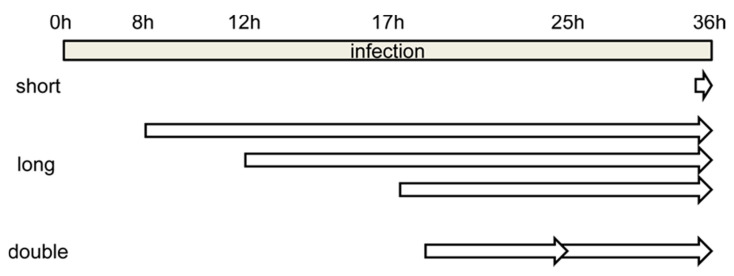
Schematics showing the time frame for the different BrdU labeling strategies tested. Short, long and double refer to incubation with BrdU for short or long time periods, or for long periods with two pulses of BrdU added (see text for details).

**Figure 3:**
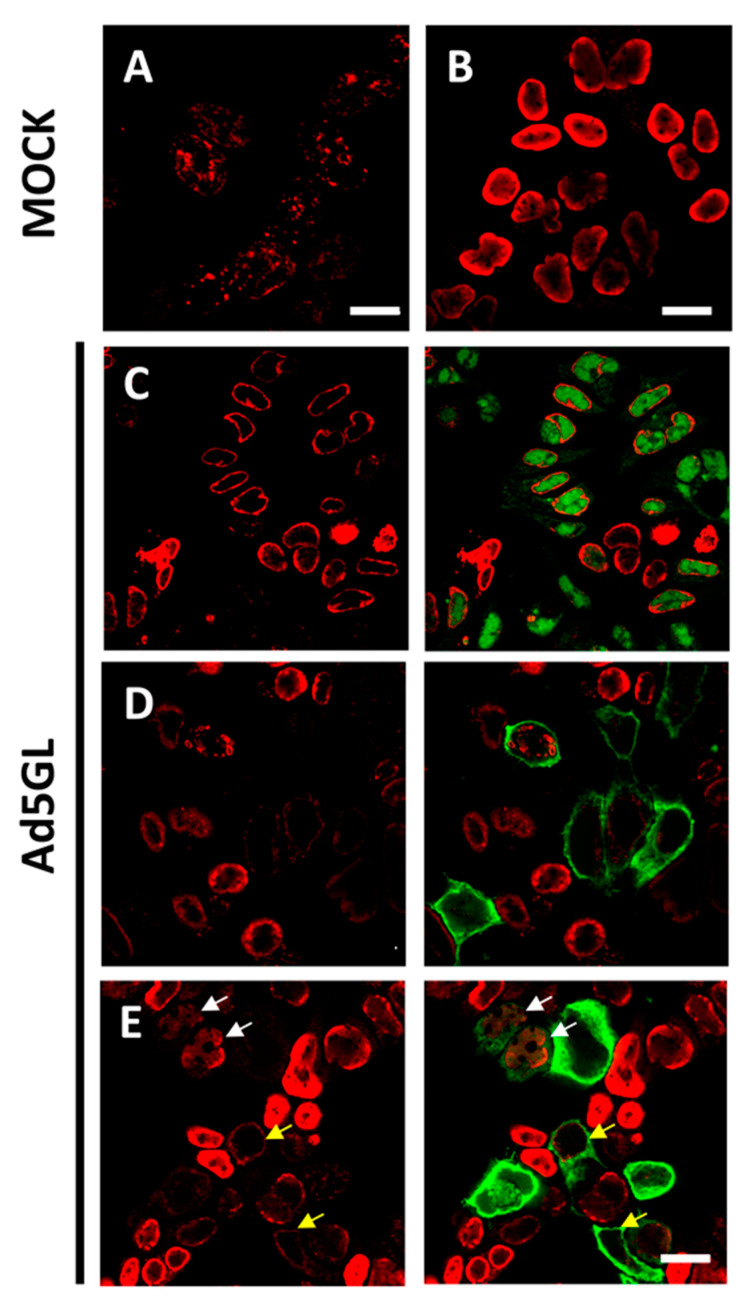
Labeling with short or long pulses of BrdU. Confocal planes showing uninfected HEK293 cells labeled 30 min before harvesting (**A**) or labeled for 24 h (**B**); and Ad5GL infected cells observed at 36 hpi with BrdU (25 µg/mL) added at (**C**) 8 hpi, (**D**) 12 hpi, or (**E**) 17 hpi. Red: BrdU. Green: GFP expressed by Ad5GL. Scale bar 10 µm in (**A**), 20 µm in (**B**–**E**). Yellow arrows indicate infected cells where the BrdU signal is similar to that in uninfected cells. White arrows indicate cells with both GFP and moderately strong BrdU signal.

One of the most used strategies in the literature is BrdU labeling for 30 min before the infection time of interest (Figure 2, short). The recommended BrdU concentration for confocal microscopy is 25 µg/mL [47]. When we added BrdU at this concentration to AdV (Ad5GL unless otherwise noted) infected cells 30 min before reaching 36 hpi, BrdU signal was detected only in uninfected cells. This signal presented a punctate pattern distributed throughout the whole nucleus (Figure 3A). This result indicates that the DNA replication rate in uninfected cells is higher than in infected cells at 35–36 hpi. Sohn and Hearing [48] observed signal in HeLa cells infected with AdV type 5 using this strategy, even employing lower amounts of BrdU (3 μg/mL). In this case, they harvested the cells at 20 hpi. According to Halbert, Cutt and Shenk [19], the AdV genome replication rate reaches the maximum value at 16 hpi, and from then on it begins to fall. This could explain why a short pulse of BrdU is enough for labelling at 20 hpi but not at 36 hpi. The AdV genome replication rate may not be high enough to incorporate BrdU in detectable amounts when the compound is added for only 30 min at 36 hpi. 

Other authors had previously observed AdV genome label in infected cells at late times of infection with short BrdU pulses, but they were using concentrations up to 15 mg/mL [9]. In studies on vaccinia virus, signal was observed in infected cells after incubation with BrdU (25 µg/mL) during 6.5 h [47]. Therefore, to overcome the low fluorescence signal obtained with short BrdU pulses at 25 µg/mL, two possible strategies could be tested: adding BrdU at earlier times of infection, or increasing the BrdU concentration.

### 3.2. Optimizing the Pulse Time: Labeling with Long BrdU Pulses

When BrdU (25 µg/mL) was added to cells infected with Ad5GL (MOI = 5) at 8 hpi, and the cells were harvested at 36 hpi (Figure 2, long), we observed a strong signal at the nucleus periphery (Figure 3C). In fluorescence confocal images, the GFP signal indicates which cells are infected. In some infected cells, the GFP signal was apparently enclosed by the BrdU nuclear signal (Figure 3C). This observation indicates diffusion of the GFP into the nucleus, as well as the large increase in nuclear volume and drastic reduction of the cytosol produced by the infection. GFP diffusion into the nucleus has previously been reported [49]. The reason why this diffusion happens more readily in some of our samples is unclear. The BrdU label at the nucleus periphery was observed both in infected (cells with GFP signal in Figure 3C) and uninfected (Figure 3B, and cells without GFP signal in Figure 3C) cells, although in the latter BrdU signal was more homogenously distributed in the nuclei. To interpret this result, we considered that host DNA is pushed towards the edge of the nucleus as the infection progresses [31]; and at 8 hpi, AdV genome replication is starting to accelerate, but shutoff of cell genome replication is not achieved yet [19,27]. Therefore, we concluded that most of the BrdU signal observed does not correspond to viral genomes, but to the host. To decrease the labelling of cellular DNA, we decided to incorporate the BrdU at later times post-infection. Additionally, the MOI was increased to 10 to be sure that all the cells were infected from the beginning. Viral particles are produced from 24 hpi, so if a monolayer is not completely infected between 24 and 36 h, some viral particles could produce and infect cells that had not been initially infected (second round of infection). Then, at 36 hpi, we would have cells at different times of infection, all of them with GFP signal. For this reason, it is important to ensure the total infection of the monolayer at the beginning of the experiment.

Increasing the MOI to 10 did not achieve the goal of having all cells infected from the beginning, as indicated by the large proportion of cells without GFP signal (Figure 3D). As for the DNA label, when BrdU was added at 12 hpi, little or no signal was observed in infected cells (Figure 3D, cells with GFP signal). This result indicates a drastic reduction in cell genome replication at 12 hpi. However, BrdU incorporation into viral genomes is not enough to produce a strong label, even if the cells are not harvested until 36 hpi. A similar result was observed when BrdU was added at 17 hpi, although in this case, some infected cells with moderately strong signal were observed (Figure 3E, white arrows). This observation indicates that BrdU incorporation into viral genomes is more effective at 17 than at 12 hpi, which is in agreement with previous reports showing that the AdV genome replication rate peaks at 16 hpi [19]. The question remained why—if the cells are not harvested until 36 hpi, leaving them time to incorporate the BrdU molecules in the medium to any newly synthesized DNA—the signal is so poor in infected cells. One possibility is that uninfected cells in the sample sequester large quantities of BrdU when their genomes replicate, competing with the infected cells. This would explain why in infected cells there is so little label when the BrdU is added at 12 hpi: when these infections reach the maximum viral replication rate at 16 hpi, there would be little or none BrdU available in the medium.

We then reasoned that, if indeed BrdU is depleted by uninfected cells, two actions were needed to solve the problem: (1) increase the MOI further to ensure that most cells in the plate were infected, and (2) add an extra dose of BrdU at about half the time between adding the first one and harvesting the cells. 

### 3.3. Optimizing the BrdU Dose: Long Pulses with Two Doses of BrdU

**Figure 4:**
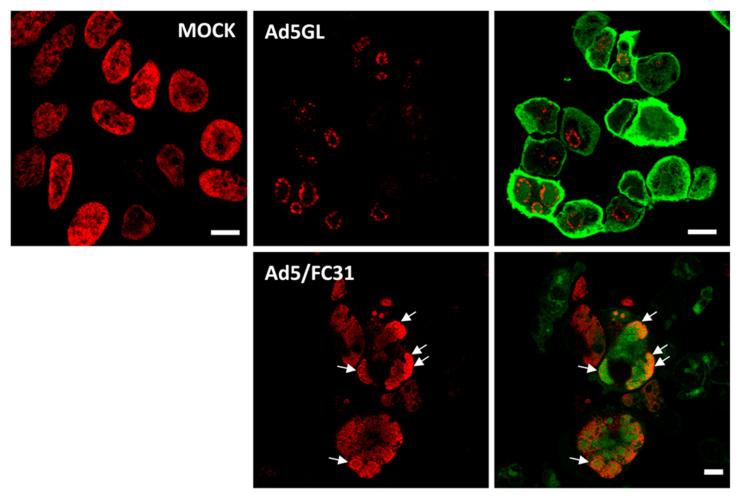
Labeling with two doses of BrdU. Confocal planes showing HEK293 cells uninfected (MOCK) or infected with Ad5GL or Ad5/FC31 at MOI = 50, as indicated. BrdU (25 µg/mL) was added first at 18 and then at 25 hpi. The cells were observed at 36 hpi. Red: BrdU, green: GFP labeled with anti-GFP antibodies in Ad5GL, GFP expressed by the virus in Ad5/FC31. See Section 3.5 for details about GFP labeling. White arrows point to concentric rings. Scale bar 10 µm.

**Figure 5:**
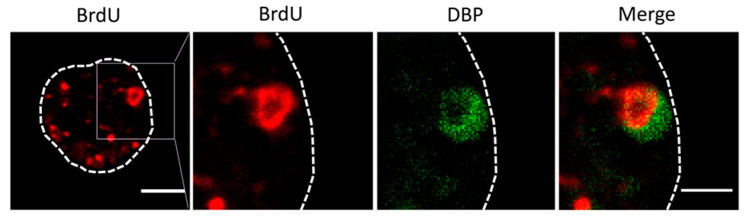
Double labeling for BrdU (red) and DBP (green) in cells infected with Ad5/FC31. The dotted line indicates the cell contour, and the square points out the magnified area in the following panels. Bar: 5 μm (left panel) and 2.5 μm (right panel).

For the next series of experiments, the cells were infected with MOI = 50, and BrdU (25 µg/mL) was added at 18 and 25 hpi (Figure 2, double). The time for adding the first dose was chosen because at 17 hpi some infected cells still presented a label pattern associated with host DNA replication (label in the nuclear periphery, Figure 3E yellow arrows). At 18 hpi, shutoff of host genome replication is well under way [27]. In these conditions, most cells were infected, and the BrdU signal distribution was different from the previously observed ones, both in infected and non-infected cells (Figure 4). The BrdU label was not confined to the nuclear periphery, but was distributed throughout the nucleus in control cells. In infected cells, a pattern of small rings (5–7 µm in diameter) was observed [27]. Double labeling with an antibody against the AdV ssDNA binding protein (DBP), the most widely used indicator of the DAS region in the AdV replication center, confirmed that these rings correspond to the PRZ, the peripheral region in the replication center where continuous genome synthesis occurs (Figure 5) [8,27]. We then tested the same protocol with the delayed packaging mutant Ad5/FC31. This mutant has the same genome replication and protein expression dynamics as the wildtype virus, but particle production is delayed by 20 h. At 36 hpi, when Ad5 reaches its particle production peak, Ad5/FC31 produces mostly empty shells [50]. Using two doses of BrdU, the accumulation of unpackaged genomes at 36 hpi in the cell was clearly observed (Figure 4). The signal was much stronger than in Ad5GL infected cells, and formed not only rings, but also concentric rings (Figure 4, white arrows), suggesting labeling of several waves of DNA synthesis in the same replication center.

### 3.4. Optimizing the BrdU Concentration: Effect of BrdU at High Concentration in Electron Microscopy

After setting up the optimal BrdU labeling conditions for immunofluorescence microscopy, we moved on to electron microscopy for a more detailed vision of the AdV late replication center. In EM, the amount of epitopes available for antibody binding is much lower than in FM. This is so, in part, because epitopes may be damaged due to chemical fixation, dehydration and resin embedding. Mostly, however, low epitope availability is due to the fact that only those sites exposed on the surface of an ultrathin (70 nm) section are accessible to the antibody molecules. This is a tiny part of all the epitopes contained in a ~20 μm diameter cell. Taking this consideration into account, for our first EM preparations, the infection and BrdU addition were carried out in the same conditions used for FM (MOI = 50, two doses of BrdU added at 18 and 25 hpi), but the BrdU concentration was increased from 25 µg/mL to 15 mg/mL, trying to maximize the amount of epitopes present in the preparations. This concentration had previously been used in EM assays in pulses of 5, 10 and 140 min [8,9].

**Figure 6:**
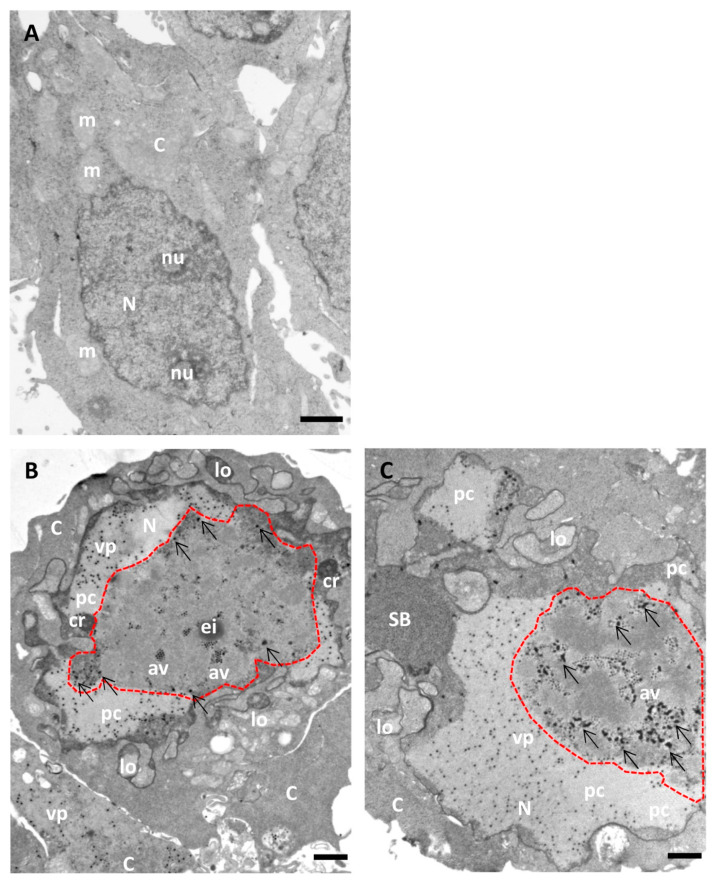
Morphology of AdV-infected cells without BrdU incorporation (for comparison with Figure 7). HEK293 cells uninfected (**A**) or infected with Ad5GL (**B**) or Ad5/FC31 (**C**) in the absence of BrdU. Cells collected at 36 hpi. MOI = 5 (similar structures were observed with MOI = 50). Nucleus (N), nucleolus (nu), cytoplasm (C), lobes (lo), compact ring (cr), electro dense inclusion (ei), protein crystal (pc), array of virus particles (av), viral particles (vp), speckled body (SB), mitochondria (m), replication center (red dotted line), and electron-dense granules (arrows). Scale bars 1 μm.

**Figure 7:**
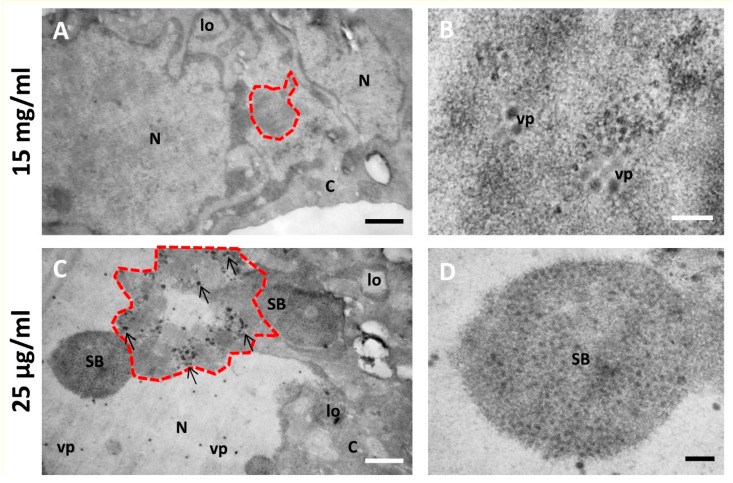
Effect of different BrdU concentrations on the morphology of AdV-infected cells. (**A**,**B**) Cells infected with Ad5/FC31 and incubated with two doses of BrdU at 15 mg/mL. (**C**,**D**) Cells infected with Ad5/FC31 and incubated with two doses of BrdU at 25 µg/mL. Cells collected at 48 hpi, MOI = 5 (similar structures were observed at MOI = 50). Nucleus (N), cytoplasm (C), lobes (lo), viral particles (vp), speckled body (SB), replication center (red dotted line), and electron-dense granules (arrows). Scale bars 800 nm (**A**,**C**) and 200 nm (**B**,**D**).

Figure 6 and Figure 7 show a comparison of Epon-embedded cells infected with Ad5GL or Ad5/FC31 in the absence or in the presence of BrdU. In the absence of BrdU, infected cells showed the modifications described in the literature, including lobulations of the nuclear envelope, compact rings, electron-dense inclusions, protein crystals, virus particles isolated or in arrays, speckled bodies, electron-dense granules, and late replication centers (Figure 6B,C) [27]. In the presence of 15 mg/mL BrdU, however, no clear AdV replication centers were observed; at most, only small condensations that could be incipient replication centers were detected (Figure 7A, red dotted line). Virus particles were notably scarce (Figure 7B). We then tested the effect of BrdU at 25 µg/mL, the concentration used for FM. In these conditions, replication centers, viral particles, and other infection-induced modifications such as SBs were readily found, and in general there were little differences with cells processed for EM in the absence of BrdU (Figure 7C,D) [27]. These results indicate that high concentrations of BrdU interfere with the establishment of the AdV replication center.

Not only were high concentrations of BrdU deleterious for AdV infection, but it has also been reported that BrdU is toxic for mammalian cells [51]. In the publications where this high concentration (15 mg/mL) was used, labeling had consisted of short pulses, or incubations spanning a maximum of two hours [8,9], vs. 18–24 h in our assays. Therefore, we conclude that BrdU at high concentration is suitable for short or moderately long pulses (less than 2 h), but for longer incubations lower concentrations of the nucleoside analog should be used.

**Figure 8:**
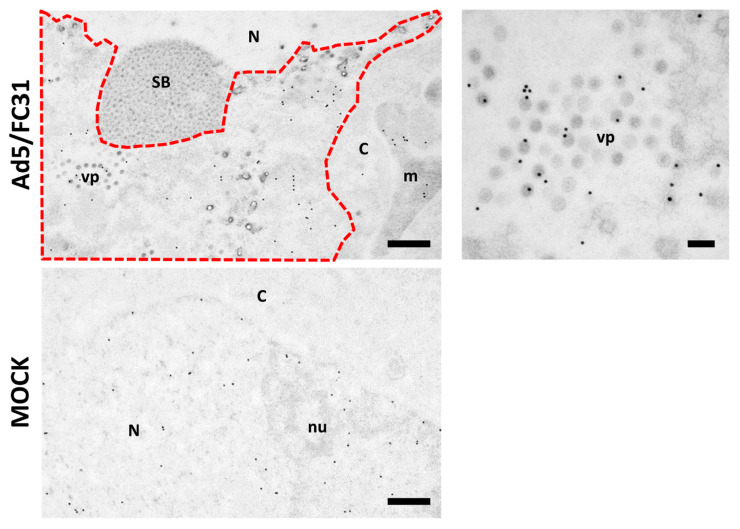
BrdU labeling for freeze-substituted samples. HEK 293 cells infected with Ad5/FC31 (MOI 50), or uninfected, as indicated. BrdU (25 µg/mL) was added at 18 and 25 hpi. The cells were fixed at 48 hpi. Nucleus (N), nucleolus (nu), cytoplasm (C), viral particles (vp), speckled body (SB), mitochondria (m), replication center (red dotted line). Scale bar: 500 nm in the left panels, 100 nm in the right hand side panel.

Freeze-substitution and immunolabeling of control and infected cells with 25 µg/mL added at 18 and 25 hpi showed that this concentration was adequate not only for preservation of cell metabolism and infection development, but also for localization of viral genomes [27]. In immunoelectron microscopy, labeling is achieved by the use of antibodies conjugated to colloidal gold particles of a defined diameter, which appear as high contrast black dots in the images. In infected cells, BrdU signal was detected in the replication center and viral particles. In uninfected cells, the BrdU signal was homogenously distributed throughout the nucleus (Figure 8). 

### 3.5. Note Regarding HCl Treatment

A harsh treatment with HCl (see Section 2) is required to denature the dsDNA so that the BrdU epitope is accessible to antibodies. This treatment poses some problems that need to be taken into account. For example, in our assays we used viruses expressing GFP, which is ideal to identify infected cells in confocal microscopy. However, the HCl treatment damages the GFP signal, and we had to resort to labeling with anti-GFP antibodies to recognize infected cells (Figure 4). HCl treatment also hindered DAPI staining of DNA, which did not provide reliable information when used in combination with BrdU labeling. In electron microscopy, HCl treatment caused tearing of the formvar support in the grids. In our experience, this problem was reduced when the plastic was deposited on the dull side of nickel grids, instead of the shiny side.

## 4. Discussion

When looking to label newly synthesized dsDNA viral genomes in infected cells, one needs to plan depending on the particular objective of the experiment, and use the previous knowledge about the infectious cycle to optimize the chances of obtaining useful, interpretable data. A fundamental issue is that BrdU will incorporate to all new DNA molecules, whether they are of viral origin or not. Therefore, it is critical to find conditions in which cell genome replication is stopped or minimized. In AdV infections, the cellular replication does not turn off completely, but it is drastically reduced from 8 hpi [19,27]. This is one of the reasons why we observed label at the nucleus periphery in infected cells when a single BrdU dose was added at 8 hpi (Figure 3C). Considering the previous knowledge regarding the virus genome replication kinetics is also of importance. Adding the labeling compound when the viral replication is at its maximum increases the likelihood of obtaining a strong signal. Short pulses at low BrdU concentration (3 μg/mL) provided label of AdV genomes in FM when the compound was added at 20 hpi [48], but not when we used 25 μg/mL at 36 hpi (Section 3.1). These results correlate with studies showing that the rate of AdV genome replication peaks at 16 hpi [19], and then begins to fall. 

Further parameters to consider are the concentration of BrdU and duration of the incubation times. Short pulses with high BrdU concentrations (15 mg/mL) have been successfully used to localize AdV genomes in EM [8,9], but the same BrdU concentration in long pulses was harmful to the cells and interfered with the infectious cycle, to the point that AdV replication centers were mostly absent in cells infected for 48 h and incubated with 15 mg/mL BrdU for 30 h (Figure 6 and Figure 7). Morris [51] compiled information on the genetic toxicity of BrdU in mammalian cells, showing that the nucleoside analog can induce sister chromatid exchange, specific locus mutations, inhibition of cell proliferation, and expression of fragile sites. The incorporation of BrdU in the genome of mammalian cells is linearly proportional to concentration. Therefore, it is necessary to be careful with the doses of this compound. Moreover, very long incubations, even at low BrdU concentration, also inhibit infection: in assays where AdV-infected cells were incubated with 30 µg/mL of BrdU for 70 h, a decrease in the production of infectious and physical virus particles was observed, but no change in cytopathic effect [14]. These authors also found that nuclear changes proceeded more slowly in the presence of BrdU, requiring about 70 hpi to reach nuclear changes similar to those observed at 48 hpi in the absence of BrdU. However, long incubations (48–72 h) with BrdU at 4–5 µg/mL have successfully been used to produce AdV particles packaging labeled genomes [52,53]. The possibility that BrdU is depleted in the medium during the course of the experiment must also be taken into account. This depletion may occur if the incubation times are very long, or if there is a large amount of cell genome replication due to the use of low MOI conditions. BrdU depletion can be prevented by adding several BrdU doses at low concentration.

Optimizing all the described factors allowed us to localize AdV genomes in late replication centers, which was crucial to identify these centers as the viral assembly factory [27]. Of course, the optimal conditions will vary depending on the viral system studied, and testing different parameters will be inescapable. We hope this account of the process and rationale followed will help other researches embarking in viral genome labeling projects for both FM and EM.

## Figures and Tables

**Table 1:** Conditions tested for BrdU labeling. IF: immunofluorescence microscopy; EM: electron microscopy: FS: freeze-substitution. Short or long indicate the length of BrdU pulse.

Experiment	MOI	BrdU Concentration	Time of BrdU Incorporation		Harvest Time (hpi)
			First Dose (hpi)	Second Dose (hpi)	
IF (short)	5	25 µg/mL	35.5	---	36
IF (long 1)	5	25 µg/mL	8	---	36
IF (long 2)	10	25 µg/mL	12	---	36
IF (long 3)	10	25 µg/mL	17	---	36
IF (2 doses)	50	25 µg/mL	18	25	36
EM-Epon 1	5 and 50 ^1^	---	---	---	48
EM-Epon 2	5	15 mg/mL	18	25	48
EM-Epon 3	5	25 µg/mL	18	25	48
EM-FS	50	25 µg/mL	18	25	48

^1^ This experiment showed that there were no differences in the cell modifications induced by infection with MOI 5 or 50.

**Table 2:** Troubleshooting table for BrdU of labeling adenovirus genomes in the cell.

Section	Problem Observed	Possible Reason	Solution
3.1	BrdU signal in uninfected but not in infected cells.	DNA replication rate was higher in uninfected than in infected cells. As a result, BrdU was only incorporated into the genome of uninfected cells.	Add BrdU when the viral genome replication rate is higher.
3.2	BrdU signal in the periphery of infected cell nuclei, in a pattern similar to that of non-infected cells.	1. BrdU was added before host genome replication was shut off. 2. MOI was not enough to ensure infection of the majority of the cells at the beginning of the experiment. Labeling reflects genome replication in cells that remained uninfected until a first virus generation was produced.	1. Add BrdU at later time post infection, when host genome replication is minimized. 2. Increase the MOI to make sure that all cells are infected from the beginning.
3.2 and 3.3	Poor signal in infected cells although BrdU was added when viral genome replication peaks.	The BrdU pulse was too long, so the compound was depleted over time.	Add a second dose of BrdU during the infection.
3.4	When incubated with BrdU, the typical adenovirus-induced nuclear modifications are not observed in EM sections of infected cells.	BrdU concentration or/and pulse duration affected cell metabolism or infection development.	Test lower BrdU concentration or shorter pulses.
3.5	Only for GFP-expressing adenoviruses: poor GFP signal in infected cells after BrdU labeling procedure.	HCl treatment used to denature DNA impaired GFP fluorescence emission.	Use antibody against GFP.
3.5	Formvar tearing in the EM grids.	HCl treatment used to denature the DNA damaged the formvar.	Coat the nickel grids by depositing formvar on the dull side rather than on the shiny side.
